# Quantitative Analysis of Calcium Spikes in Noisy Fluorescent Background

**DOI:** 10.1371/journal.pone.0064394

**Published:** 2013-05-31

**Authors:** Radoslav Janicek, Matej Hotka, Alexandra Zahradníková, Alexandra Zahradníková, Ivan Zahradník

**Affiliations:** 1 Institute of Molecular Physiology and Genetics, Slovak Academy of Sciences, Bratislava, Slovakia; 2 Department of Biophysics, Faculty of Natural Sciences, Pavol Jozef Šafárik University, Košice, Slovakia; University of Maribor, Slovenia

## Abstract

Intracellular calcium signals are studied by laser-scanning confocal fluorescence microscopy. The required spatio-temporal resolution makes description of calcium signals difficult because of the low signal-to-noise ratio. We designed a new procedure of calcium spike analysis based on their fitting with a model. The accuracy and precision of calcium spike description were tested on synthetic datasets generated either with randomly varied spike parameters and Gaussian noise of constant amplitude, or with constant spike parameters and Gaussian noise of various amplitudes. Statistical analysis was used to evaluate the performance of spike fitting algorithms. The procedure was optimized for reliable estimation of calcium spike parameters and for dismissal of false events. A new algorithm was introduced that corrects the acquisition time of pixels in line-scan images that is in error due to sequential acquisition of individual pixels along the space coordinate. New software was developed in Matlab and provided for general use. It allows interactive dissection of temporal profiles of calcium spikes from x-t images, their fitting with predefined function(s) and acceptance of results on statistical grounds, thus allowing efficient analysis and reliable description of calcium signaling in cardiac myocytes down to the *in situ* function of ryanodine receptors.

## Introduction

Calcium signaling in cardiac myocytes has high dynamics requiring sub-micron and sub-millisecond resolution for exact characterization. Calcium signals can be observed by means of calcium-sensitive fluorescent indicators and confocal microscopy either as calcium sparks [1] or as calcium spikes [2], which differ in the way they are measured. Both types of signals represent local calcium release events due to activation of ryanodine receptors in dyadic clusters [3] that are typically about 0.2 µm in diameter.

Calcium sparks are measured in intact cardiac myocytes loaded with the indicator in the membrane-permeable esterified form, or in patch-clamped myocytes dialyzed with the indicator directly. Calcium sparks can be well discerned only when activation of calcium releasing sites occurs with a low frequency (typically <20 s^−1 ^per 100 µm of scanned cell length [4]); otherwise sparks merge into macrosparks [4], waves [4], [5] or whole cell calcium transients [6]. For this reason they are acquired in recordings lasting many seconds. Calcium sparks represent transient local increases of fluorescence intensity proportional to the instantaneous local concentration of calcium ions [6]. Automatic algorithms were developed that detect sparks in confocal images as statistically significant local increases of fluorescence [4], [7]–[10]). Of major practical use is the frequency of spark occurrence per unit volume, which is sensitive to experimental interventions [11] and is related to functionality and density of calcium releasing sites [12], [13]. Deriving the calcium release flux from the calcium spark requires the use of mathematical models that include reaction-diffusion properties of the cytosol [14]. Phenomenologically, however, the time course of sparks can be well described by a sum of two sequential exponential processes [15]. Still, sparks are usually characterized by descriptors such as amplitude, full width at half-maximum, time to peak, and full duration at half-maximum [4], [7]–[10]). The spatial size of calcium sparks is relatively large. Measured at half-maximum they are 1.75–2 µm wide [4], [5]. This is the length of a typical sarcomere or twice the average distance between release sites in three dimensions [16]. This large size, together with duration of about 30 ms [4]) makes resolving neighboring release sites difficult and complicates interpretation of the data.

Calcium spikes are measured in patch-clamped myocytes dialyzed with the calcium-sensitive indicator and the calcium chelator EGTA, which buffers small increases of calcium and thus limits calcium signals to vicinity of an activated calcium release site [2]. Under these conditions, individual release sites can be discerned from each other even at maximal stimulation [2], [17]. The small spatial size of the spikes allows the use of low aperture pinholes to effectively reduce interference of the out-of-focus events by increasing the spatial resolution in the z-direction. The amplitude of calcium spikes is proportional to the intensity of the local calcium release flux [2], [18], which allows direct observation of the kinetics of calcium release and of its relationship to the triggers [17], [19]. This advantage has not yet been fully exploited. The reason is that calcium spikes are difficult to describe due to their rapid kinetics and inherently small signal-to-noise ratio.

**Figure 1:**
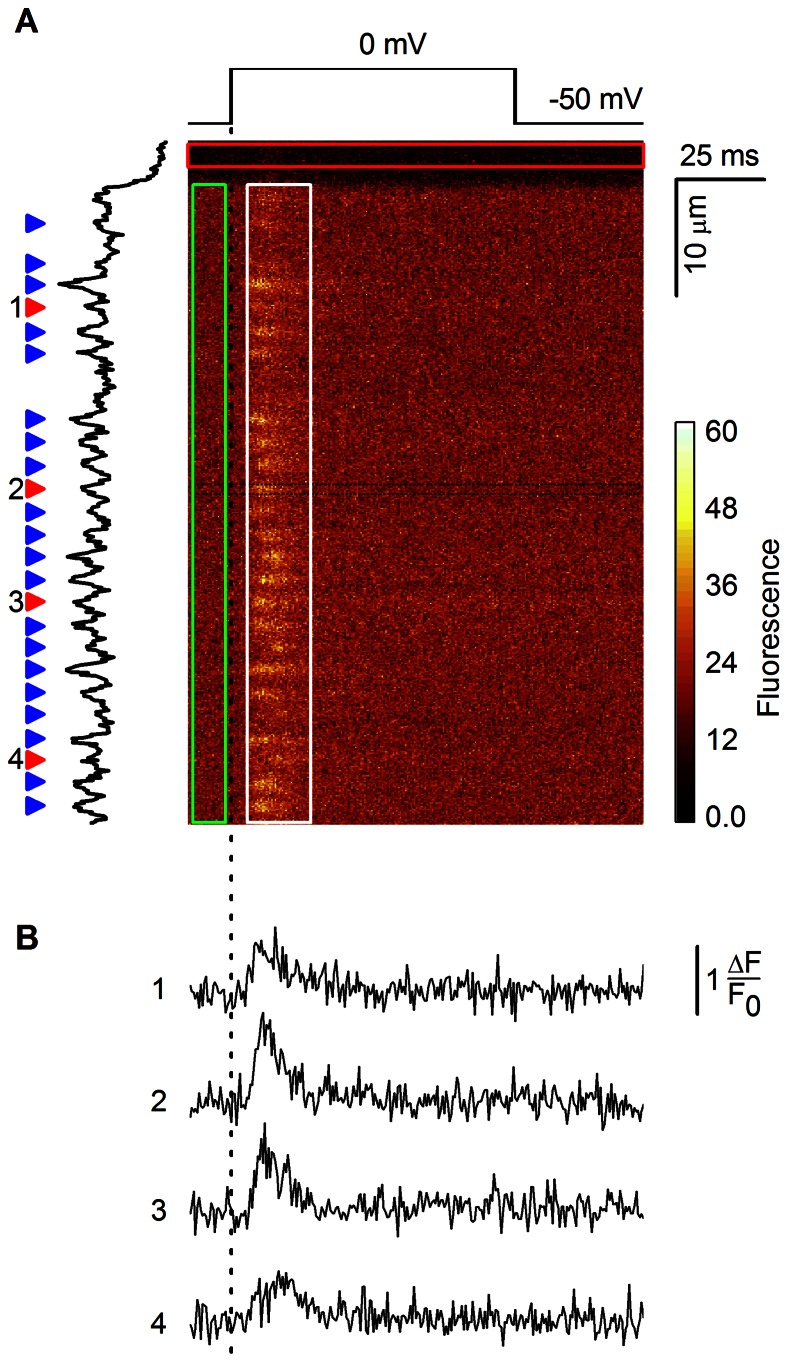
Detection and extraction of calcium spikes for analysis. A: a line scan confocal (x-t) image with three indicated regions of interest. The fluorescence intensity is color-coded. The red rectangle delineates the non-cell region used for estimation of the background fluorescence level. The green rectangle delineates the scan region preceding the stimulus (shown above the image) that is used for estimation of baseline cell fluorescence and the level of noise. The white rectangle delineates the region of image chosen for calcium spike detection. The black trace at the left represents the temporal average of fluorescent intensity of the pixels in the white rectangle. The arrowheads mark calcium spikes selected for analysis. B: The time courses of the fluorescent profiles of the spikes marked by the numbered red arrowheads in A. The traces were obtained by averaging 7 spatial pixels delineated by the thin black lines indicated around the calcium spike #2. The time scale is the same for A and B.

The confocal images of calcium spikes provide spatial information in the x axis, temporal information in the y axis, and the calcium flux information in the fluorescence intensity values (Figure 1). Correct detection and characterization of calcium spikes is not trivial. Many technical aspects were analyzed and solved previously [2], [18]. Recently we realized that in laser scanning confocal microscopy measurements, the differences in the time of acquisition of individual image pixels that arise due to sequential principle of the line scan are not taken into account. This can be a source of a serious error when it comes to the study of very fast and brief time-variable processes. Additionally, algorithms developed to analyze calcium sparks do not detect calcium spikes reliably. Application of general methods of increasing signal to noise ratio (SNR), such as signal or image filtration techniques, substantially changes the kinetics of calcium spikes. Therefore, we have introduced a method based on fitting of the noisy fluorescent traces containing calcium spikes with a mathematical model of the time course of calcium spikes [18]. This allowed characterization of calcium spikes under various experimental manipulations [17], [19]. However, the precision of parameter estimation and the detectability of calcium spikes with a given SNR had not been determined. It had been originally assumed that the amplitude distribution of calcium spikes, measured using the low-affinity calcium indicator Oregon Green BAPTA 5-N, was biased by the existence of out-of-focus spikes, in analogy to calcium sparks [7]. The putative low amplitude events would have a low detection probability; thus, a large fraction of spikes has been considered undetected [18]. On the other hand, the amplitude distribution of spikes with a high SNR obtained using Fluo-3, a high affinity calcium indicator, indicated a very low fraction of undetected events, as if the most of detected spikes were in focus and their amplitude distribution was not distorted due to missed events [19]. This discrepancy asks for assessment of the limits of calcium spike detectability.

In this work we characterize the relationship between the signal-to-noise ratio and the quality of parameter estimation of calcium spikes. This was made effective by original spike analysis software that included correction for pixel acquisition time implemented in the MATLAB environment. We conclude that fitting the time course of the spikes by a kinetic model allows reliable detection and quantitative description of calcium spikes even at the lowest experimentally observed SNRs, which together with their excellent spatio-temporal resolution and straightforward interpretation favors calcium spikes over calcium sparks in studies aimed to clarify calcium signaling in cardiac myocytes.

## Methods

The analysis procedure developed in this study was tested on the dataset of images of calcium spikes recorded in isolated rat cardiac myocytes described previously [17]–[19]. We tested the analysis using calcium spikes recorded either with Oregon Green BAPTA-5N (OG-5N) or Fluo-3 differing in the rate of calcium binding and in the fluorescence signal-to-noise ratio. All anaesthetic and surgical procedures were approved by the State veterinary and food administration of the Slovak Republic and by the Ethical Committee of the Institute of Molecular Physiology and Genetics, Slovak Academy of Sciences.

Calcium spikes of the OG-5N or Fluo-3 indicator type were simulated with parameter values set to mimic experimental records and used to design, test and optimize the analysis procedures.

### Acquisition, Extraction and Fitting of Calcium Spikes

In experiments, the calcium spikes were recorded and extracted as illustrated in Figure 1. A part of an isolated whole-cell patch-clamped cardiac myocyte was scanned by an excitation laser in a direction parallel to the longitudinal axis (vertical axis in the image) at a selected frequency. The emitted fluorescence light was collected by a confocal microscope equipped with a photomultiplier and its output signal was digitized. In this way, one column of pixels was recorded at each scan. The resulting x-t images were corrected for the average amplitude of the background signal estimated from the region not containing the cell (Figure 1A, red rectangle). Positions of the identified calcium spikes were marked and the time-intensity traces of calcium spikes were obtained by averaging 7 spatial pixels (0.81 µm) centered at the maximum of the spike to reduce noise. The spike intensity was expressed as fluorescence increase, ΔF, normalized to the mean baseline fluorescence level, F_0_, estimated in the interval preceding the voltage stimulus (green rectangle in Figure 1A). The traces were accepted for analysis if the amplitude of the putative spike passed the user-specified signal-to-noise-ratio (SNR) threshold. This subjective step could lead to acceptance of false positive spikes and to rejection of smaller calcium signals with obvious consequences for the validity of spike statistics. The fluorescence traces (Figure 1B) were then fitted with the model spike function Eq. 1, which is an abbreviated form of the original function [18]:
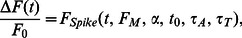
(1)where Δ*F* = *F*(*t*) – *F*_0_ is the fluorescence difference, *F_0_* is the baseline fluorescence, *F_Spike_* is the theoretical time course of the spike, *t* is the time elapsed from the start of the voltage stimulus, *t_0_* is the latency of the onset of calcium spike relative to the onset of the voltage stimulus, *F_M_* is the maximal increase of *F*/*F*_0_ in the absence of release termination, α is the fraction of the signal contributed by calcium concentration build-up [2], and *τ_A_* and *τ_T_* are the time constants of spike activation and termination, respectively. The full format of Eq. 1 is given in the supplemental data (Methods S1, Eq. S1). For simplicity, we have not considered traces with two spikes in this work; nevertheless, the principle of analysis would be the same [19].

**Figure 2:**
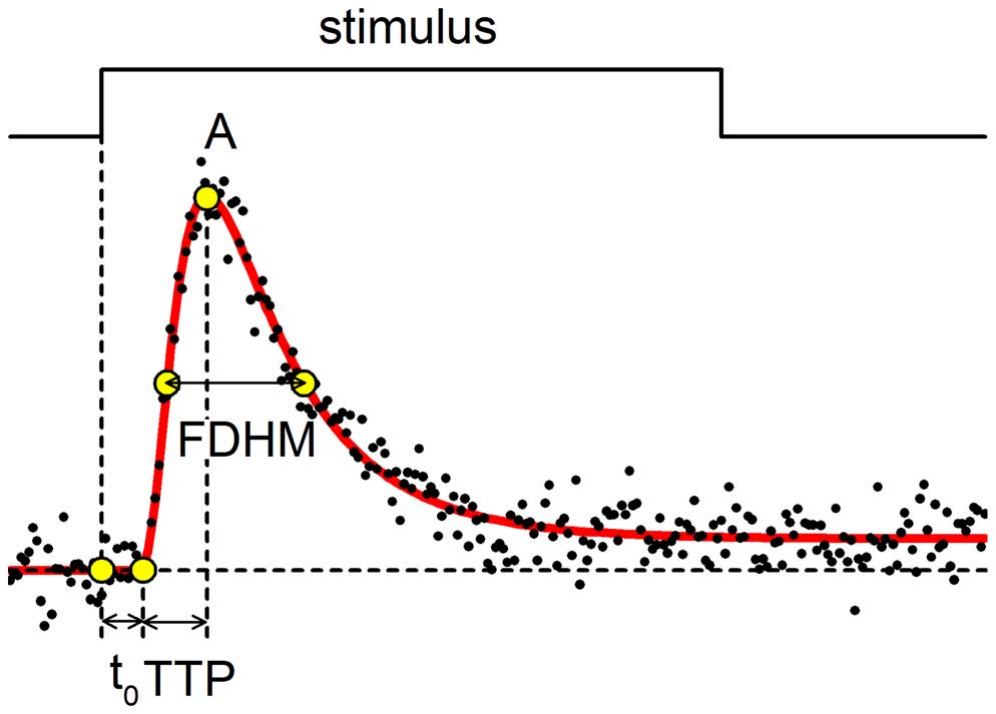
Description of calcium spikes. Voltage stimulus from −50 to 0 mV is shown in the top panel. The extracted time course of a calcium spike (black dots) was fitted with the theoretical function (red line, Eq. 1). The latency t_0_ was estimated by fitting, and the descriptors were estimated numerically as illustrated. A – peak amplitude, t_0_–latency, TTP – time to peak, FDHM – full duration at half maximum.

Since the kinetic model of calcium spikes is phenomenological and its parameters, except for latency (t_0_), may not have direct mechanistic meaning [18], the spikes were characterized by t_0_ and by the descriptors numerically determined from the theoretical traces simulated with the fitted parameters (Figure 2), specifically: peak amplitude (A), time to peak (TTP, the interval between the onset and the peak of a spike), and full duration at half-maximum (FDHM, the interval between time points for half-maximal amplitudes at the ascending and the descending arm of a fitted spike).

### Simulation of Synthetic Calcium Spikes

Three dataset types of synthetic spikes were generated using Eq. 1 (Table S1 and Table S2 in File S1). The first type mimicked experimental spikes for the indicators OG-5N and Fluo-3. The spike parameters were varied randomly and independently around their mean respective values published previously [18]. A collection of 1000 spikes was generated for each indicator, and a random instance of noise was added to each spike. These datasets were used to find the optimal fitting algorithm, to test the equivalence of the fitting procedure with the original interactive procedure, and to test the validity of the results.

The second dataset type consisted of traces containing only pure noise. A collection of 1000 traces was generated. This dataset was used to test the fitting algorithms for their tendency to identify false-positive spikes.

The third dataset type was created by combining one simulated spike for each indicator with 1000 different instances of noise to test the effect of noise on the accuracy and precision of parameter determination. The parameters of Eq. 1 used for spike simulation assumed the mean values for each indicator, taken from [18]. Seven datasets were generated for OG-5N with SNR of 1, 1.5, 2, 3, 5, 7 and 10; and nine datasets for Fluo-3 with SNR of 1.0, 1.5, 2.0, 3.0, 5.0, 7.0, 10, 15, and 20).

The noise consisted of normally distributed pseudorandom numbers, generated using the Mersenne twister algorithm [20], with the mean equal to zero and the standard deviation defined as:
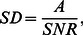
(2)where A is the mean peak amplitude of fluorescence increase taken from [18] and SNR is the required signal-to-noise ratio.

### Statistical Analysis

Statistical analysis was performed in Origin (Ver. 8 SR 6). The significance of differences between fits was determined using the F-test and p = 0.05 as criterion.

### SpikeAnalyzer

To speed up and automate the analysis of large sets of confocal x-t images, the SpikeAnalyzer software was developed as a Windows standalone application built in the MATLAB Compiler (Ver. 7.17 (R2012a), Mathworks, USA). The logical scheme of data processing and the description of individual analysis steps are given in Software Description S1. In brief, the analysis is configured using the headers of the confocal image file (the Leica TIFF file format) and of the corresponding electrophysiological record file (the Axon ABF file format) or manually for incompatible file formats. Individual calcium spikes are selected by the user and fitted by Eq. 1. The best fits of all selected traces are displayed for evaluation and eventual refitting of individual traces if needed. The results of analysis are saved as an XLS project for further use. The source code of the SpikeAnalyzer and the compiled program are deposited under the Academic Free License (http://opensource.org/licenses/AFL-3.0) at sourceforge.net (http://sourceforge.net/projects/spikeanalyzer/?source=navbar).

## Results

This study focuses on practical aspects of calcium spike description. These include the correction of pixel acquisition time for analysis of confocal laser scanning images, the selection of an appropriate fitting algorithm, the analysis of the accuracy and precision of calcium spike parameter estimation in large groups of variable spikes such as those observed in experiments, and of the effect of signal-to-noise ratio on the detectability of spikes and estimation of their parameters.

### Correction of Pixel Acquisition Time

**Figure 3:**
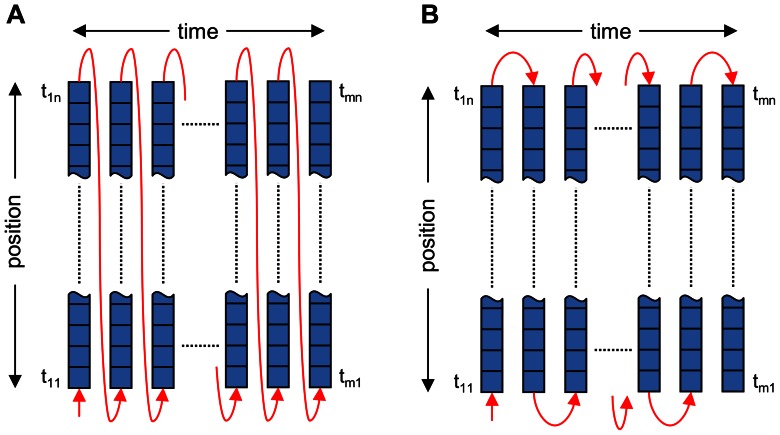
The temporal sequence of pixels in an image acquired in the x-t line scan mode. Panels A and B describe the unidirectional and the bidirectional mode, respectively, as rendered in the confocal image, that is, each pixel in a line is assigned the same acquisition time. In fact, each subsequent pixel is shifted along the time axis by the value of the pixel integration time, and each line is shifted by the scanner reversal time. Red arrows indicate oscillations of the scanner. The squares represent the acquired pixels of the confocal image; t_ij_ is the time of acquisition of the pixel in the i^th^ column and j^th^ row of the image (*i = *1 … *m*, *j = *1 … *n*), described by Eq. 3 or Eq. 4 for the unidirectional or the bidirectional mode.

Images generated by laser scanning confocal microscopes are digital representations of the output signal of a single photomultiplier responding to the flux of photons collected by the confocal microscope. The photons are emitted by fluorophores excited by the laser beam that scans the sample. When the fluorescence is not constant in time, as in the case of calcium signals or moving objects, the scanning principle introduces systematic errors, since the image pixels corresponding to different positions are acquired at different times (Figure 3). Pixelization, that is, digitization of the photomultiplier output, is synchronized with the scanner running either in unidirectional or bidirectional scanning mode. In either case, after scanning a line, the scanner has to stop and reverse its sweep direction, which needs considerable time. Depending on the scanning mode, the pixel acquisition of the next line starts either in a reverse order relative to the preceding line, or in the same order but only after another reversal of the sweep direction (Figure 3). As a result, the neighbor pixels within lines are separated by time intervals equal to the pixel integration time, and the neighbor pixels in columns are separated by a line acquisition time. The line acquisition time is incremented regularly in the unidirectional mode and periodically varying in the bidirectional mode. Taken together, the pixel acquisition time in the image depends on the line frequency, the number of pixels per line, and the scanning mode. This may introduce a substantial error in estimation of kinetic parameters of time-variable processes and has to be corrected in exact experiments.

To estimate the actual time of acquisition of a pixel we have used a light emitting diode driven by TTL signals of the digitizer (Axon Digidata 1440A, Axon Instruments, USA) and controlled by the pClamp software (Ver. 10, Axon Instruments, USA). The light pulses from the diode were recorded by the confocal microscope in each scanning mode and their images were used for exact determination of the pixel integration time. The results for x-t scanning modes and 512 pixels per line at individual line frequencies are given in Table 1.

**Table 1:** The measured pixel integration times at different scanning modes and frequencies (Leica TCS SP2 AOBS).

Scan speed	Scanning mode	Scanning frequency	Pixel integration time
(Hz)		*f* (Hz)	*t_p_* (µs)
400	unidirectional	400	1.39
400	bidirectional	800	
800	unidirectional	800	0.658
800	bidirectional	1600	
1000	unidirectional	1000	0.515
1000	bidirectional	2000	

The actual time of acquisition of individual pixels of the image was expressed as a matrix with elements corresponding to the time of individual pixels using Eq. 3 and Eq. 4 for the unidirectional and bidirectional mode, respectively:
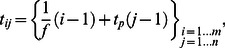
(3)and
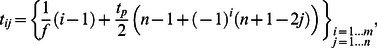
(4)where tij is the time value of an element in the ith row and the jth column of the image matrix, f is the line scanning frequency, tp is the pixel integration time, m is the number of lines per image, i is the line number, n is the number of pixels per line, and j is the pixel number in the line. It should be noted that in the bidirectional mode the pixel acquisition time increase with j for odd rows and decrease with j for even rows of the image (Eq. 4).

In the standard procedure without correction, the pixel acquisition times of spikes, that is, positions of samples on the time axis, were calculated using Eq. 5:
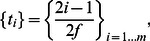
(5)where *t_i_* is the time of acquisition assigned to all pixels on the i^th^ scanning line, which corresponds to the time of acquisition of the 1^st^ pixel in the line, *f* is the line scanning frequency, *i* is the line number and m is the number of lines in the x-t image.

### The Effect of Sequential Sampling on Spike Parameter Estimation

Sequential sampling of spatial pixels may introduce a significant error in estimation of calcium spike timing that varies with the position of spikes in the image. This error can be calculated if the time of acquisition of individual pixels of the image is known. When with the correction of pixel acquisition time the assigned time of a pixel acquisition in line *i* at position *j* is *t_ij_* (Eq. 3 or 4) and without correction it is *t_i_* (Eq. 5), then the position time error is *t_pte_* = *t_ij_* – *t_i_* for unidirectional scanning and *t_pte_* = (*t_ij_*+*t_ij_*_+1_)/2– (*t_i_*+*t_i_*_+1_)/2 for bidirectional scanning. However, in images obtained by bidirectional scanning, an additional systematic temporal error at a given spatial position arises due to the alternation of the acquisition time increments (Figure 3), which varies with the spatial position of a pixel.

The effect of the correction of pixel acquisition time on the estimated values of spike parameters was determined using noise-free simulated spikes with 10 different latencies (3.9–4.4 ms) that spanned the whole interval of one line-scan sampling period. The spikes were localized to the left-side, center, and right-side positions of the image at *i* = 4, 256, and 509, respectively. These positions allowed the standard 7-pixel averaging of fluorescence signals and estimation of the extent of the position dependent time error. The resulting 30 simulated spikes were sampled at pixel acquisition times corresponding to 1000 Hz (unidirectional) or 2000 Hz (bidirectional) line frequency with and without correction for pixel acquisition time. Each trace was fitted and the obtained parameters were compared with their respective seeded values. The comparison revealed that if the correction for pixel acquisition time was applied, all spike parameters were determined correctly for both scanning modes (not shown). Without the correction, all parameters but the latency were also determined correctly for both scanning modes (not shown). The error of latency determination was independent of the spike onset relative to the time of sampling (ANOVA, p = 0.38 for unidirectional and p = 0.99 for bidirectional scanning). However, it was dependent on the position of the spike on the spatial axis (Table 2).

**Table 2:** Errors of latency estimation without correction of pixel acquisition time.

Scanning method	Spike position (pixel)	predicted error t_i_ - t_ij_ (µs)		estimated error t^fit^ - t_0_ (µs)
		Odd lines	Even lines	
unidirectional	4	1.546	1.546	1.533±0.007
	256	131.442	131.442	132.573±0.007
	509	261.854	261.854	264.125±0.006
bidirectional	4	1.546	261.854	140.16±0.17
	256	131.442	131.958	132.811±0.0001
	509	261.854	1.546	140.43±0.17

The effect of the left to right position of the spike in the image on the error in latency estimation can be characterized by the difference between the pixel acquisition time estimated without (*t_i_*) and with the correction (*t_ij_*). The question is whether the same difference in latency will be estimated by fitting the spikes. Analysis of the fitting results for both scanning modes revealed that the difference between the latency determined by fitting and the seeded latency value, t^fit^ - t_0_, was very close to the (*t_i_* - *t_ij_*) values and reached up to 26.4% of the line scanning period (Table 2). In the case of bidirectional scanning, the position time error at odd lines combined with the position time error at even lines, therefore, the difference in fitted latencies was close to the average of the respective pixel acquisition times at the given position on the spatial axis (Table 2).

In our previous studies we used a 7-pixel averaging to reduce the noise fluctuations. To determine the error introduced by sequential sampling in the case of the 7-pixel averaging, we used analogous procedure as that described above (note that no noise was added in these simulations). We found that spike parameters, including latency, estimated using 7-pixel averaging were not significantly different from those estimated using only the central pixel (not shown). In other words, the sequential error in the case of spatial averaging was the same as if the signal occurred only at the central pixel.

### Testing of Fitting Algorithms and Procedures

The performance of MATLAB minimization algorithms in estimation of calcium spike parameters was tested using the dataset of the first type (see Methods) with a white noise level of 0.15 RMS. Each parameter value was randomly generated around a mean value and with a standard deviation in correspondence to those of experimental spikes recorded with the OG-5N indicator (Table S1 in File S1).

The effects of minimization algorithm, robustness of method, and constraining of the fitted parameters on the quality of spike description were explored. Four minimization algorithms were available in MATLAB: Nelder-Mead simplex, Trust-Region, Levenberg-Marquardt and Gauss-Newton (Table S3 in File S1). Of these, Trust-Region, Levenberg-Marquardt and Gauss-Newton could be used either without a robust method, or with the LAR or Bisquare robust methods; The Levenberg-Marquardt and Gauss-Newton algorithms did not handle constraints on fitted parameters. In total, there were 14 possible combinations of minimization algorithm, robustness of method, and constraining of the fitted parameters, which were evaluated according to the fraction of fits that provided either acceptable spike parameters, that is, physically acceptable parameter values (Selection A: τ_A_ ≥1 ms, τ_T_ ≥1 ms, FDHM <40 ms, 0 ms ≤ t_0_<80 ms, A ≥0.01 F/F_0_) or “detectable” amplitude (Selection B: A>SNR), or a statistically acceptable fit, that is, a statistically significantly better than the fit with a constant.

According to all criteria, the Trust Region and Simplex algorithms with parameter constraining and without robust method performed the best. For unconstrained parameters, the performance of the algorithms was Simplex>Levenberg-Marquardt>Trust Region>Gauss-Newton. All algorithms provided a larger fraction of acceptable fits under constrained than under unconstrained conditions (for the Trust Region: 95.1 vs. 77.5%; for Simplex: 95.1 vs. 90.1%). The use of either LAR or Bisquare robust methods reduced the fraction of acceptable fits substantially and independent of constraints and algorithm.

The correlation between the fitted and the simulated parameters for spikes that passed the F-test was excellent in all cases. Individual fitting procedures were ranked according to the average correlation coefficient of the descriptors A, t_0_, TTP and FDHM in Table S4 in File S1. The best correlations were achieved by the Trust Region algorithm with constrained parameters (R = 0.986, 0.893, 0.869 and 0.942 for A, t_0_, TTP and FDHM, respectively). With unconstrained parameters, the Levenberg-Marquardt algorithm performed the best (R = 0.981, 0.891, 0.855 and 0.929 for A, t_0_, TTP and FDHM, respectively). The algorithms using the LAR robust method provided the worst correlations (R = 0.907–0.935, 0.770–0.798, 0.728–0.775 and 0.843–0.887 for A, t_0_, TTP and FDHM, respectively). For the latency, the correlation between the simulated and fitted values was the best for the Simplex with constrained parameters (R = 0.904), while the algorithms with the LAR robust method were the worst (R = 0.770–0.798).

Individual fitting procedures were ranked according to the fraction of outliers in any parameter. The fraction of outliers was relatively small (≤2%) and occurred mostly among estimates of A, FDHM and TTP. The unconstrained Trust Region algorithm provided the smallest fraction of outliers (0.52%). In the case of latency all algorithms and methods provided very low fraction of outliers (0–0.3%). The fraction of fitted parameter values outside the range of their mean ±3 × s.d. is given in Table S5 in File S1.

For the purpose of automatic analysis, an optimal fitting algorithm should provide a low probability of false positive spikes. A dataset of the second type (see Methods) with a white noise level of 0.15 RMS was analyzed, and when a putative spike was detected by the algorithm, the validity of the result was tested. All algorithms detected a significant number of false-positive spikes in the pure noise data traces. When the constrained version of the algorithms was used, false-positive spikes were detected in all traces. None of the false-positive spikes for any algorithm and constraints was accepted by the F-test (p = 0.05), that is, for each trace a fit with a constant was better. Notably, a significant proportion (up to ∼60%) of these false spikes would be acceptable on the basis of their parameter values (τ_A_ >1.0 ms, τ_T_ >1.0 ms, FDHM <40 ms, 0≤ t_0_<80 ms, A ≥0.01 F/F0) or “detectable” amplitude (A>SNR), see Table S6 in File S1.

In conclusion, the best results were obtained, i.e., the spike parameter estimation process was optimal, when fitting was performed with constraints on parameter values and the acceptance of spikes was based on the result of the F-test. Under these settings, false positive detection of spikes was fully avoided. The fitting algorithms that did not use robust parameter estimation were clearly preferable. The constrained Trust Region algorithm scored best in all tests of parameter estimation except for correlation between simulated and fitted latency, in which it was the second best after Simplex. However, since the relative error in latency estimation was significantly smaller in the Trust Region algorithm (0.06±0.61% vs. 2.13±0.61%; p = 0.02), it was selected as the method of choice for further evaluations. The constrained Simplex algorithm achieved the best correlation between simulated and fitted latency and performed the second best in the remaining tests.

### Test of the Equivalence between the Interactive and the Automatic Fitting Procedures

To verify the equivalence between the interactive fitting procedure using Origin, applied in our previous studies, and the automatic MATLAB procedure applied in SpikeAnalyzer, we used again the synthetic dataset simulating the OG-5N spikes used previously for selecting the optimal fitting algorithm, since it offers the most challenging task due to its low SNR. Simulated spikes with noise were analyzed either by the constrained Trust-Region algorithm in MATLAB (without any robust method) or by the constrained Levenberg-Marquardt algorithm in ORIGIN (the same constraints were used in both programs) since the constrained fitting with the Levenberg-Marquardt algorithm used in our previous studies was not implemented in MATLAB and the Trust Region algorithm that performed best in MATLAB was not implemented in Origin. The initialization vector was set to the mean of the experimentally observed values ([18], Table S1 in File S1). The lower and upper bounds were [0< t_0_< ∞, 0< F_M_<∞, 1< τ_A_<∞, 1< τT<∞, 0< α <1].

**Figure 4:**
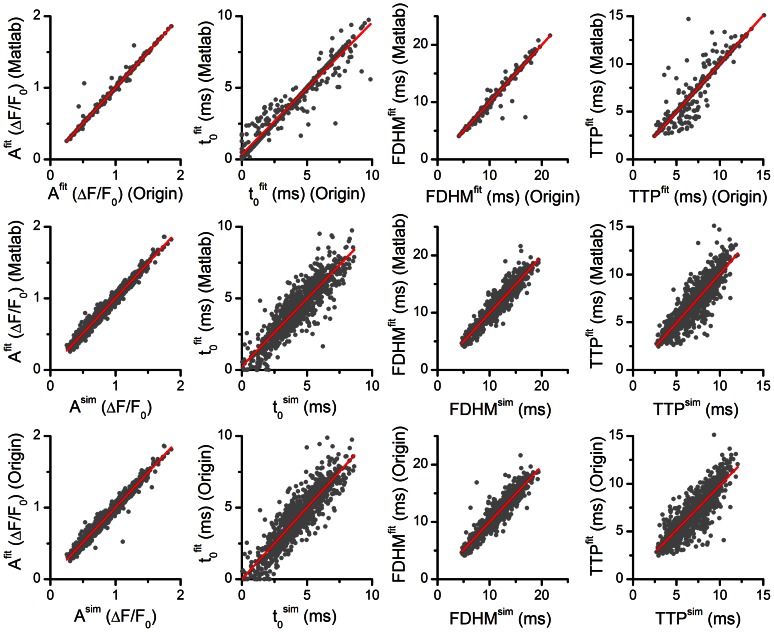
Comparison of the parameter values estimated by different fitting procedures. The Levenberg-Marquardt algorithm in Origin and the Trust-Region algorithm implemented in MATLAB were used on a dataset of 1000 simulated spikes with white noise and normally distributed parameter values. Top row – correlation between parameter values estimated by fitting simulated spikes in Origin and in MATLAB. Middle row – comparison of the fitted parameter values obtained in MATLAB with the input parameter values used in simulations. Bottom row – comparison of the fitted parameter values obtained in Origin with the input parameter values used in simulations. Red lines are linear fits (correlation coefficients are given in Tables S4 in File S1. It should be noted that a majority of the data points lie exactly under the regression lines.

It turned out that regarding the fraction of accepted spikes, both procedures provided similar results. The correlations between input parameters and the results of fitting, as well as between the results of SpikeAnalyzer and ORIGIN are shown in Figure 4 and in Table S4 in File S1. Considering only the spikes that passed the F-test, the SpikeAnalyzer procedure provided more precise estimates of the amplitude, FDHM and TTP, while ORIGIN provided a more precise estimate of the latency of spikes; however, the differences were not substantial. The correlation between the SpikeAnalyzer and ORIGIN parameter estimates was very good for all spike parameters (R = 0.996, 0.972, 0.954 and 0.990 for the values of A, t_0_, TTP and FDHM, respectively). In both, the automatic and the interactive procedures, the estimated peak amplitude values were marginally but systematically higher (p<0.001) than the input values by 0.006 and 0.005 ΔF/F_0_ units on average (a mean relative error of +0.7% and +0.6%, respectively). Other parameters were not significantly different from the input parameters.

In conclusion, the automatic procedure implemented in the SpikeAnalyzer provides results equivalent to those of the original interactive analysis procedure [18].

### Accuracy of Spike Parameter Estimation

The adequacy of the theoretical spike fitting function (Eq. 1) for description of the time course of calcium spike fluorescence was demonstrated previously [18]. Nevertheless, the accuracy and precision of the spike parameter estimation have not been rigorously examined. Here we used a dataset of synthetic spikes that emulated the experimental data obtained for OG-5N and Fluo-3 indicators and the SpikeAnalyzer procedure. In other words, the level of noise and the distribution of spike parameters were the same as experimentally determined, thus giving rise to the experimentally observed distribution of signal-to-noise ratios. The synthetic dataset of 1000 spikes with OG-5N characteristics was the same as the one used for testing the fitting procedures (see above). Spike parameters had normal distributions with mean and standard deviation taken from Zahradnikova et al. [18]. The average and minimum values of the SNR for the synthetic datasets were 5.39±2.11 and 1.67, respectively, for OG-5N and 14.19±5.60 and 1.85 for Fluo-3, respectively.

In the case of simulated OG-5N spikes the fitting procedure succeeded in 95.1%; that is, the remaining 4.9% of fitted spikes did not pass the F-test (see Table S3 in File S1). In the case of simulated Fluo-3 spikes the fitting procedure succeeded in 99.6% and 0.4% of fitted spikes did not pass the F-test. The fitted spikes that did not pass the F-test were mostly those with low SNR (<2.5). Two spikes with SNR of 2.7 and 3.5 were rejected despite good accordance between the simulated and the fitted parameters; thus, the probability of incorrect rejection of spikes by the F-test (p = 0.05) was ∼0.2%.

**Figure 5:**
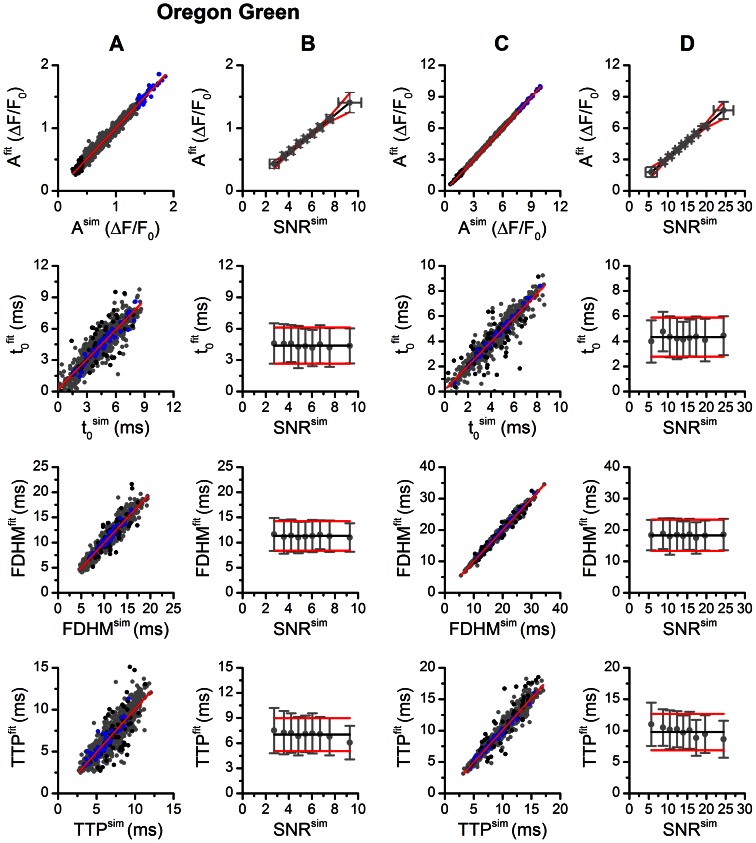
Determination of the accuracy and the precision of the estimated parameters of OG-5N (A, B) and Fluo-3 spikes (C, D). **A, C** – correlation between the simulated and the fitted parameters (grey circles). Red line is the linear fit. Parameters of the spikes with the bottom 5% of SNR values (2.38±0.04 for OG-5N and 4.33±0.15 for Fluo-3) and of spikes with the top 5% of SNR values (10.11±0.12 for OG-5N and 26.68±0.29 for Fluo-3) are shown as black and blue circles, respectively. Pearson correlation coefficients between the parameters of simulated OG-5N calcium spikes and parameters of their fits were 0.986, 0.893, 0.869 and 0.942 for the A, t_0_, TTP and FDHM values, respectively. Pearson correlation coefficients between the parameters of simulated Fluo-3 calcium spikes and parameters of their fits were 0.999, 0.939, 0.962 and 0.993 for the A, t_0_, TTP and FDHM values, respectively. **B, D** – mean ± s.d. of the fitted parameter values grouped by the SNR values of the simulated spikes. Black lines are the mean parameter values of the simulated spikes in the absence of noise. Red lines show mean+s.d. and mean - s.d. of parameter values of noise-free simulated spikes.

The correlations between the simulated and estimated parameters are shown in Figure 5A, C and the dependence of the estimated parameter values on SNR is shown in Figure 5B, D. The comparison of data points corresponding to spikes with the lowest and the highest signal-to-noise ratio shown in Figure 5A, C revealed that, first, the whole range of parameter values was present in all SNR bands, and, second, that the quality of correlation between the simulated and estimated parameters decreased with decreasing SNR but remained acceptable even at the lowest SNR (p<10^−6^, F test). Despite considerable uncertainties in estimation of spike parameters at small SNR, the overall correlations between the simulated and estimated parameters were excellent (p = 0, F-test). The distributions of the estimated parameter values at individual SNR levels (circles with error bars) are compared to the mean and standard deviation (black line and red lines) of the parameters of the simulated noise-free spikes in Figure 5B, D. In the whole examined range the distribution of the estimated parameters followed the distribution of the simulated parameters. This indicates that the quantitative uncertainties in parameter estimation are caused by a random contribution of noise to their exact values.

In conclusion, estimation of spike parameters based on the mathematical model (Eq. 1) provides reliable results for both, OG-5N and Fluo-3 spike data. Since only a small fraction of synthetic spikes could not be detected and analyzed reliably (0.5% in the case of Fluo-3 spikes and 5% in the case of OG-5N spikes), the problem of missed spikes is not crucial in real experiments.

### Limitations of the Fitting Procedure at a Low Signal-to-noise Ratio

The Trust Region fitting algorithm used in SpikeAnalyzer was further validated by analyzing simulated synthetic spikes that differed only in the level of noise. A dataset of 1000 spikes was created by combining a simulated spike, one for Fluo-3 and one for OG-5N, with 1000 different noise traces with defined values of SNR. The parameters of the simulated spikes were assigned the mean values found in real experiments (Table S1 and Table S2 in File S1). We created seven datasets for OG-5N (SNR of 1, 1.5, 2, 3, 5, 7 and 10, respectively) and nine datasets for Fluo-3 (SNR of 1, 1.5, 2, 3, 5, 7, 10, 15, and 20, respectively). The initialization vector for the fitting procedure was equal to the parameter values used in simulations. The fits that did not pass F-test were not accepted and further analyzed.

**Figure 6:**
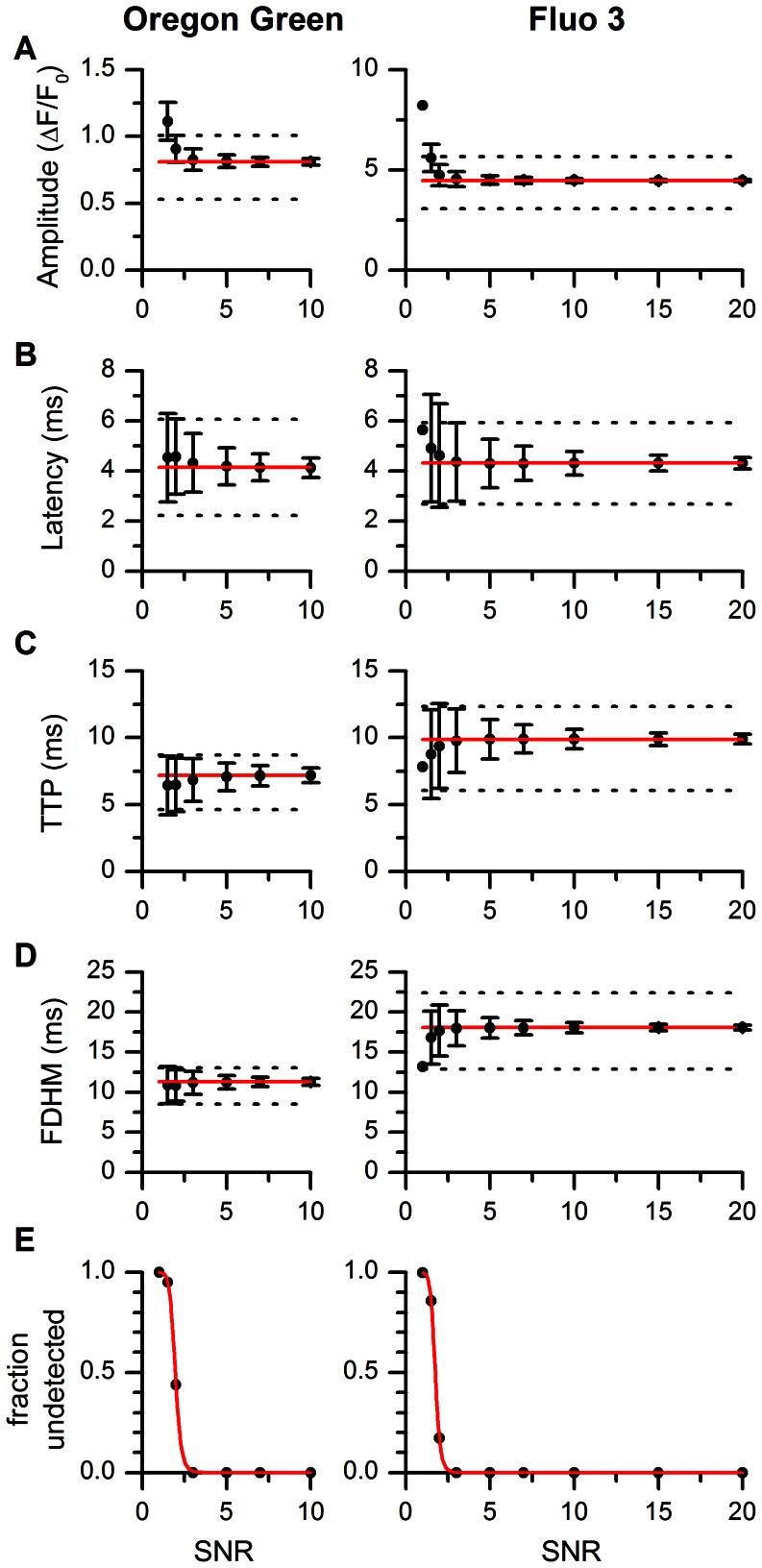
Tests of accuracy of the estimated parameters of OG-5N and Fluo-3 spikes. A-D: Estimated parameters of OG-5N and Fluo-3 spikes in the datasets with different SNR values. The values of spike parameters obtained by fitting are plotted as mean ± s.d. Red solid lines are the parameter values used for simulation of spikes. Dashed lines are the mean+s.d. and the mean – s.d. parameter values estimated experimentally ([18]). Note the different scaling of y axes in A. The data points for SNR of 1 in Fluo-3 column are without dispersions as they represent a single detected spike. E: The fraction of undetected spikes. Red solid lines are fits with the logistic curve *y* = 1/(1+ (*x*/*S_5_*_0_)*^n^*) where *S_50_* and *n* are 1.96 and 11.3, respectively, for OG-5N spikes, and to 1.75 and 11.7, respectively, for Fluo-3.

The results of analysis are summarized in Figure 6. It is apparent that for the lowest SNR levels corresponding to those in real experiments (SNR = 2 for OG-5N and SNR = 5 for Fluo-3, see Figure 5), the differences in the mean parameter values caused by the noise are comparable or lower than the experimental dispersion of the parameters. For the average and high SNR levels, the errors introduced by the noise are much lower than the dispersion of the parameters. A significant fraction of undetected spikes (>5%) occurred only at SNR ≤2.5 in both, OG-5N spikes and Fluo3 spikes (Figure 6E). Based on the published statistics ([18], see Table S1 and Table S2 in File S1), such low values of SNR occur in 8% of spikes in OG-5N and in 2% of spikes in Fluo-3. Thus, the fraction of undetected spikes is low in the whole range of SNR values pertinent to experiments. For both indicators, the spike amplitudes were slightly overestimated at the lowest SNR values (Figure 6A), for reasons not well understood.

## Discussion

A well-recognized obstacle in the study of local calcium signaling is the high noise of calcium spikes [17]–[19]. Therefore, studies from other laboratories limited the analysis to characterization of the average fluorescence transient of calcium spikes [2], [3], [21]–[23] and to estimation of the fraction of active junctions [22], [23], and only few analyzed peak amplitudes [21], [22], [24] and durations at half maxima of individual spikes [24]. The estimation of spike latency depends strongly on the detection method, which were based either on the amplitude threshold or on fitting. Therefore, the differences in latencies estimated under similar conditions in different laboratories are much larger than the differences observed upon experimental manipulation (cf. [17]–[19] and [22]). Exact estimation of this parameter is crucial for understanding the coupling between the L-type DHPR calcium channels and the calcium-releasing RyR channels.

In the present study we have developed new semiautomatic software for analysis of calcium spikes. In comparison with the previous interactive method, an increase in the image analysis throughput by an order of magnitude was achieved. Additionally, the software obviates the manual data transfer between different programs, which virtually eliminated mistakes in data handling and greatly enhanced the data processing productivity.

### Accuracy and Precision of Parameter Estimation

The control over the fitting routines in the MATLAB environment enabled us to assess the accuracy and precision of the descriptive parameters of calcium spikes. There were large differences in the ability of individual algorithms and methods to converge to valid solutions, which resulted in different fractions of fitted spikes acceptable by thee F-test (Table S3 in File S1). When converging, individual algorithms did not produce parameters significantly different from the true ones, but differed slightly in the accuracy and precision of parameter estimation. For adequate performance at the experimentally achieved signal-to-noise ratios, the algorithms should not employ the robust fitting methods, and parameters should be constrained. The use of the F-test for assessing the statistical significance of the fitting results proved essential for avoiding detection of false positive spikes and for correct acceptance of spikes at low SNR values.

The evaluation of the accuracy and precision of parameter estimation at different levels of noise provided two important insights: First, in simulated spikes with parameters distributed according to the experimental data, the dispersion of fitted parameters was comparable to the dispersion of input parameters. Since the results of the new method were equivalent to those of the old method, this indicates the reliability of parameter estimates determined in previous studies [17]–[19]. Second, in simulated spikes that had identical parameters but variable SNR, the accuracy was relatively independent of SNR, and the relative error of parameter estimation increased above 10% only at SNR values below 2, which is rarely present in real experiments (0.5% of data in Fluo-3 and 5.4% of data in OG-5N). Additionally, even at SNR = 1.5 the dispersion of the fitted parameters was close to the experimentally observed dispersion at higher SNR values (Figure 6A–D).

### Comparison with Alternative Procedures

Overall, procedures implemented in SpikeAnalyzer performed reasonably well with respect to alternative procedures of calcium signal analysis. For instance, 50% detectability (S_50_) of calcium sparks was obtained for sparks with amplitude of 0.30 and a SNR of 0.9 [7]. Similar detection efficiencies of S_50_ = 0.7–1.1 SNR were obtained with other spark detection techniques [8]–[10]. This is about a half of the value (twice the sensitivity) estimated in this work (S_50_ = 1.7–2.0 SNR; Figure 6). It should be noted, however, that the noise of the raw images in our study was reduced by a factor of 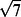
≈ 2.65 by the 7-pixel averaging, i.e., the value of S_50_ analogous to that obtained in spark analysis would be 0.64–0.76 of SNR before averaging.

The data obtained in this work provide evidence that the amplitude distribution of Fluo-3 calcium spikes reported by Zahradnikova et al. [18] and Janicek et al. [19] was not significantly distorted by undetected events, since only 0.8% of the recorded spikes had SNR <2 [19], while 85% of events should be detected at SNR = 2. This supports the claim of [19] that spike amplitude distribution is not considerably distorted by the presence of spikes below the detection limit. Calcium spikes measured using the OG-5N indicator [18] might have been incompletely detected due to the low SNR [17], [18], but as we show here (Table S3 in File S1), the fraction of undetected events was not more than 5%.

The SpikeAnalyzer procedure outperforms the spark detection algorithms in the rejection of false-positive events. At the SNR level corresponding to the S_50_, most spark detection algorithms yield a substantial proportion (10–50%) of false positive events [7]–[10]. The procedure used in SpikeAnalyzer fully suppresses the false positive detections on statistical grounds at a significance level of 0.05.

The accuracy of the estimated amplitude of calcium spikes was better than the corresponding accuracy of estimated amplitude of sparks, which was shown to be overestimated by at least 10% [7] or, in the case of events with low spatial width (FWHM <2 µm), underestimated by 24–52% [8]. In this study, the estimate of spike amplitude was higher by only 0.7%.

### Implications for Interpretation of Calcium Spikes

The limited precision of parameter estimation may be one of the sources of the normal distribution of quantal amplitudes of spikes [19] that was estimated to ∼0.6 ΔF/F_0_ units with Fluo-3 as the indicator at a SNR level of 5–10. Under similar conditions, the s.d. of the amplitude estimate in the present study was 0.1–0.2 ΔF/F_0_ units. This means that only about 15–30% of the observed dispersion in the quantal amplitudes of calcium spikes is due to the limited precision of their estimation.

### Conclusions

Although small and fast, calcium spikes can be well characterized by the fitting procedure scrutinized in this study that is capable to extract their parameters properly. Statistical analysis revealed that even when the spikes are barely visible the average values of their descriptors are correct estimates of real values. Due to their better spatial and temporal resolution, calcium spikes outshine calcium sparks for in situ studies of ryanodine receptor activity at individual calcium release sites down to the single molecule level

## Supporting Information

File S1 **Supporting Tables.** Table S1 The parameter values of simulated OG 5-N spikes. Table S2 The parameter values of simulated Fluo-3 spikes. Table S3 Ranking of the fitting algorithms according to the fraction of accepted fits. Table S4 Ranking of the fitting algorithms according to correlation between the simulated and fitted parameters. Table S5 Ranking of the fitting algorithms according to the fraction of outliers. Table S6 Ranking of the fitting algorithms according to the fraction of false positive spikes.
(PDF)

Methods S1 **The full form of Equation 1 of the main text used for fitting.** **Eq. S1**.
(PDF)

Software Description S1 **Operation of the SpikeAnalyzer program.** Screenshot S1 The Analyzer dialog window. Screenshot S2 The Fit Results Summary window. Screenshot S3 The Fitter dialog window. Screenshot S4 The FitParameters dialog window.
(PDF)
