# Supplementary material for: Quantitative Analysis of Calcium Spikes in Noisy Fluorescent Background
Source: PLoS One. 2013 May 31;8(5):e64394. doi: 10.1371/journal.pone.0064394 (PMC3669300; doi:10.1371/journal.pone.0064394)
Supplement: File S1 — Supporting Tables. Table S1 The parameter values of simulated OG 5-N spikes. Table S2 The parameter values of simulated Fluo-3 spikes. Table S3 Ranking of the fitting algorithms according to the fraction of accepted fits. Table S4 Ranking of the fitting algorithms according to correlation between the simulated and fitted parameters. Table S5 Ranking of the fitting algorithms according to the fraction of outliers. Table S6 Ranking of the fitting algorithms according to the fraction of false positive spikes. (PDF) [file pone.0064394.s001.pdf]

Quantitative analysis of calcium spikes in noisy fluorescent background

Radoslav Janicek[1], Matej Hotka[1,2], Alexandra Zahradníková, Jr. [1], Alexandra Zahradníková[1], and Ivan Zahradník[1].

[1]Institute of Molecular Physiology and Genetics, Slovak Academy of Sciences, Vlárska 5, 833 34 Bratislava, Slovakia, [2]Department of Biophysics, Faculty of Natural Sciences, Pavol Jozef Šafárik University, Košice, Slovakia

Supporting Tables

Table S1 **The parameter values of simulated OG 5-N spikes.**

Parameters and descriptors	Simulation 1 (mean ± S.D.) N = 1000	Simulation 2	Reference (mean ± S.D.) N = 144
t_0 (ms)	4.39 ± 1.72	4.13	4.13 ± 1.92
F_M	1.58 ± 0.55	1.577	1.577 ± 0.53
τ_A (ms)	3.10 ± 1.04	3.13	3.13 ± 1.32
τ_I (ms)	5.50 ± 2.10	5.48	5.48 ± 2.64
α (s^{-1})	3.75 ± 1.97	3.70	3.70 ± 0.32
A	0.82 ± 0.32	0.81	0.77 ± 0.24
TTP (ms)	11.28 ± 2.95	11.29	10.80 ± 2.28
FDHM (ms)	7.02 ± 1.98	7.19	6.67 ± 2.04
SNR	5.39 ± 2.11	5.08	5.08 ± 1.68

Simulation 1: The mean and standard deviation of parameters and descriptors in the test dataset of simulated spikes that mimicked experiments (see Reference values). The parameter values of Eq. 1 were normally distributed pseudorandom numbers, generated using the Mersenne twister algorithm [1] with Reference parameters (last column), and selected using the criteria $t_0 \geq 0$, $\tau_A > 1$ ms, and $\tau_T > 1$ ms. The values of descriptors were determined from the simulated spikes numerically before addition of noise.

Simulation 2: The set of parameters and descriptors of the single simulated spike corresponded to the mean experimental estimate.

Reference: Experimental mean and standard deviations of parameters and descriptors of calcium spikes [2].

Table S2. **The parameter values of simulated Fluo-3 spikes**.

Parameter	Simulation 1 (mean ± S.D.) N = 1000	Simulation 2	Reference (mean ± S.D.) N = 118
t_0 (ms)	4.33 ± 1.56	4.31	4.31 ± 1.63
F_M	7.52 ± 2.74	7.54	7.54 ± 2.72
τ_A (ms)	3.84 ± 1.37	3.8	3.80 ± 1.96
τ_I (ms)	10.51 ± 3.56	10.44	10.44 ± 3.80
α (s^{-1})	5.38 ± 1.64	5.37	5.37 ± 0.15
A	4.46 ± 1.76	4.47	4.36 ± 1.30
TTP (ms)	9.77 ± 2.89	9.88	9.20 ± 3.15
FDHM (ms)	18.30 ± 4.97	18.07	17.63 ± 4.78
SNR	14.19 ± 5.60	13.86	13.86 ± 5.21

Simulation 1: The mean and standard deviation of parameters and descriptors in the test dataset of simulated spikes that mimicked experiments (see Reference values). The parameter values of Eq. 1 were normally distributed pseudorandom numbers, generated using the Mersenne twister algorithm [1] with Reference parameters (last column), and selected using the criteria $t_0 \geq 0$, $\tau_A > 1$ ms, and $\tau_T > 1$ ms. The values of descriptors were determined from the simulated spikes numerically before addition of noise.

Simulation 2: The set of parameters and descriptors of the single simulated spike corresponded to the mean experimental estimate.

Reference: Experimental mean and standard deviations of parameters and descriptors of calcium spikes [2].

Table S3. **Ranking of the fitting algorithms according to the fraction of accepted fits.**

				Fraction accepted [%]		
**Rank**	**Constraints**	**Algorithm**	**Robust method**	**Selection A**	**Selection B**	**After F-test**
1	yes	TR	none	92.3	99.3	95.1
2	yes	Simplex	n.a.	92.3	99.3	95.1
3	no	Simplex	n.a.	89.2	92.8	90.1
4	yes	TR	Bi	86.3	99.3	89.2
5	n.a.	LM	none	84.3	88	85.1
6	n.a.	LM	Bi	83.3	87	81
7	no	TR	none	76.8	79.3	77.5
8	no	TR	Bi	75.6	77.6	73.4
9	no	GN	none	62.9	65	62.7
10	no	GN	Bi	61.8	62.7	59.8
11	yes	TR	LAR	88.8	99	33.9
12	n.a.	LM	LAR	80.5	86.1	29.4
13	no	TR	LAR	72.3	76.3	26.8
14	no	GN	LAR	58.9	61.3	22.4
	Original interactive method			99.8	99.2	95.0

TR – Trust Region; LM – Levenberg-Marquardt; GN – Gauss-Newton
Bi – Bisquare robust method; LAR – LAR robust method; n.a. – not applicable

Selection A: spike parameters satisfying conditions: $\tau_A > 1.000001$, $\tau_T > 1.000001$, FDHM < 40, $t_0 > 0$, $t_0 < 80$, $A \geq 0.01$

Selection B: fitted amplitude A > SNR

Original interactive method – LM algorithm in Origin

Table S4. **Ranking of the fitting algorithms according to correlation between the simulated and fitted parameters.**

				Pearson correlation coefficient				
**Rank**	**Constraints**	**Algorithm**	**Robust method**	**A**	**t_0**	**TTP**	**FDHM**	**average**
1	yes	TR	none	0.986	0.893	0.869	0.942	0.923
2	yes	Simplex	n.a.	0.986	0.904	0.859	0.942	0.922
3	yes	TR	Bi	0.985	0.895	0.862	0.939	0.920
4	no	LM	none	0.981	0.891	0.855	0.929	0.914
5	no	Simplex	n.a.	0.983	0.895	0.838	0.925	0.910
6	no	LM	Bi	0.978	0.884	0.845	0.922	0.907
7	no	TR	none	0.982	0.874	0.841	0.927	0.906
8	no	TR	Bi	0.979	0.867	0.829	0.920	0.899
9	no	GN	Bi	0.983	0.876	0.821	0.911	0.898
10	no	GN	none	0.979	0.876	0.795	0.893	0.885
11	yes	TR	LAR	0.924	0.798	0.775	0.887	0.846
12	no	GN	LAR	0.935	0.794	0.737	0.845	0.828
13	no	LM	LAR	0.907	0.792	0.749	0.851	0.825
14	no	TR	LAR	0.914	0.770	0.728	0.843	0.813
	Original method			0.982	0.899	0.840	0.931	0.913

TR – Trust Region; LM – Levenberg-Marquardt; GN – Gauss-Newton
Bi – Bisquare robust method; LAR – LAR robust method; None – no robust method; n.a. – not applicable
The highest correlation coefficients are shown in bold; the lowest correlation coefficients are shown in italics.

Table S5. **Ranking of the fitting algorithms according to the fraction of outliers.**

Rank	Constraints	Algorithm	Robust method	N	Fraction of outliers [%]
1	no	TR	none	775	0.52
2	yes	TR	Bi	892	0.56
3	no	LM	none	851	0.59
4	no	TR	Bi	734	0.68
5	no	LM	Bi	810	0.74
6	no	Simplex	n.a.	901	0.78
7	yes	Simplex	n.a.	951	0.84
8	yes	TR	none	951	0.84
9	yes	TR	LAR	339	0.88
10	no	GN	none	627	0.96
11	no	GN	Bi	598	1.00
12	no	LM	LAR	293	1.37
13	no	TR	LAR	267	1.50
14	no	GN	LAR	223	1.79

N – the number of spikes that passed the F-test
TR – Trust Region; LM – Levenberg-Marquardt; GN – Gauss-Newton
Bi – Bisquare robust method; LAR – LAR robust method; None – no robust method; n.a. – not applicable

Table S6. **Ranking of the fitting algorithms according to the fraction of false positive spikes.**

				Number of false positives			
**Rank**	**Constraints**	**Algorithm**	**Robust method**	**All false positives**	**Selection A**	**Selection B**	**After F-test**
1	no	GN	LAR	59	10	03	0
2	no	GN	Bi	69	17	05	0
3	no	LM	LAR	145	36	26	0
4	no	TR	LAR	163	31	18	0
5	no	GN	none	173	36	04	0
6	no	TR	Bi	180	38	13	0
7	no	LM	Bi	204	61	18	0
8	no	Simplex	n.a.	257	92	19	0
9	no	LM	none	284	88	21	0
10	no	TR	none	312	68	16	0
11	yes	Simplex	n.a.	1000	249	57	0
12	yes	TR	Bi	1000	289	147	0
13	yes	TR	LAR	1000	326	201	0
14	yes	TR	none	1000	596	104	0

Selection A: parameters of false positive spikes satisfying conditions: $\tau_A > 1.000001$, $\tau_T > 1.000001$, FDHM < 40, t0 > 0, t0 < 80,A $\geq$ 0.01

Selection B: fitted amplitude of false positive spikes A > SNR

The algorithms were ranked according to the number of fits that provided false-positive spikes

REFERENCES

1. Matsumoto M, Nishimura T (1998) Mersenne twister: a 623-dimensionally equidistributed uniform pseudo-random number generator. TOMACS 8: 3-30.
2. Zahradnikova A, Jr., Polakova E, Zahradnik I, Zahradnikova A (2007) Kinetics of calcium spikes in rat cardiac myocytes. J Physiol 578: 677-691.
