# Supplementary material for: Quantitative Analysis of Calcium Spikes in Noisy Fluorescent Background
Source: PLoS One. 2013 May 31;8(5):e64394. doi: 10.1371/journal.pone.0064394 (PMC3669300; doi:10.1371/journal.pone.0064394)
Supplement: Methods S1 — The full form of Equation 1 of the main text used for fitting. Eq. S1. (PDF) [file pone.0064394.s002.pdf]

Quantitative analysis of calcium spikes in noisy fluorescent background

Radoslav Janicek[1], Matej Hotka[1,2], Alexandra Zahradníková, Jr. [1], Alexandra Zahradníková[1], and Ivan Zahradník[1].

[1]Institute of Molecular Physiology and Genetics, Slovak Academy of Sciences, Vlárska 5, 833 34 Bratislava, Slovakia, [2]Department of Biophysics, Faculty of Natural Sciences, Pavol Jozef Šafárik University, Košice, Slovakia

Supporting Methods

The full form of Equation 1 of the main text used for fitting is:

For $t \le \mathrm{t}_0$: $\dfrac{\Delta F}{F_0} = y_0$; Eq. S1a

For $t > \mathrm{t}_0$:

$$\frac{\Delta F}{F_0} = y_0 + F_{Spike}(t, F_M, \alpha, t_0, \tau_A, \tau_T) = y_0 + \frac{\mathrm{F_M}}{(\tau_A - 3\tau_I)(\tau_A - 2\tau_T)(\tau_A - \tau_T)} \cdot$$

$$\cdot \left(-\frac{3}{2} e^{-\frac{2(t-\mathrm{t}_0)}{\tau_\mathrm{A}}} \tau_\mathrm{A}(2-\alpha\tau_\mathrm{A})(4\tau_\mathrm{A} - 7\tau_\mathrm{T})(\tau_\mathrm{A} - 3\tau_\mathrm{T}) + 3e^{-\frac{(t-\mathrm{t}_0)}{\tau_\mathrm{A}}} \tau_\mathrm{A}(\tau_\mathrm{A} - 3\tau_\mathrm{T})(\tau_\mathrm{A} - 2\tau_\mathrm{T}) + \right.$$

$$+ \frac{3}{5} \mathrm{e}^{-\frac{5(t-\mathrm{t}_0)}{\tau_\mathrm{A}}} \tau_\mathrm{A}(5-\alpha\tau_\mathrm{A})(2\tau_\mathrm{A} - 5\tau_\mathrm{T})(\tau_\mathrm{A} - \tau_\mathrm{T}) - \frac{1}{6} \mathrm{e}^{-\frac{6(t-\mathrm{t}_0)}{\tau_\mathrm{A}}} \tau_\mathrm{A}(6-\alpha\tau_\mathrm{A})(\tau_\mathrm{A} - 2\tau_\mathrm{T})(\tau_\mathrm{A} - \tau_\mathrm{T}) -$$

$$- 6\mathrm{e}^{-\frac{t-\mathrm{t}_0}{\tau_\mathrm{T}}} \tau_\mathrm{I}^3(1-\alpha\tau_\mathrm{T}) - \frac{3}{4} \mathrm{e}^{-\frac{4(t-\mathrm{t}_0)}{\tau_\mathrm{A}}} \tau_\mathrm{A}(4-\alpha\tau_\mathrm{A})\left(5\tau_\mathrm{A}^2 + 20\tau_\mathrm{A}\tau_\mathrm{T} + 17\tau_\mathrm{T}^2\right) +$$

$$+ \frac{1}{3} \mathrm{e}^{-\frac{3(t-\mathrm{t}_0)}{\tau_\mathrm{A}}} \tau_\mathrm{A}\left(\tau_\mathrm{A}^2(57 - 10\alpha 0_\mathrm{A}) - 3\tau_\mathrm{A}(84 - 13\alpha 3_\mathrm{A})\tau_\mathrm{T} + (249 - 29\alpha 9_\mathrm{A})\tau_\mathrm{T}^2\right) +$$

$$+ \frac{18\mathrm{e}^{-\left(\frac{1}{\tau_\mathrm{A}} + \frac{1}{\tau_\mathrm{T}}\right)(t-\mathrm{t}_0)} \tau_\mathrm{T}^3(\tau_\mathrm{A} + \tau_\mathrm{T} - \alpha\tau_\mathrm{A}\tau_\mathrm{T})}{\tau_\mathrm{A} + \tau_\mathrm{I}} - \frac{18\mathrm{e}^{-\left(\frac{2}{\tau_\mathrm{A}} + \frac{1}{\tau_\mathrm{T}}\right)(t-\mathrm{t}_0)} \tau_\mathrm{T}^3(\tau_\mathrm{A} + (2-\alpha\tau_\mathrm{A})\tau_\mathrm{T})}{\tau_\mathrm{A} + 2\tau_\mathrm{I}} +$$

$$+ \frac{\alpha(\tau_\mathrm{A} - 3\tau_\mathrm{T})(\tau_\mathrm{A} - 2\tau_\mathrm{T})(\tau_\mathrm{A} - \tau_\mathrm{T})\left(37\tau_\mathrm{A}^4 + 252\tau_\mathrm{A}^3\tau_\mathrm{T} + 605\tau_\mathrm{A}^2\tau_\mathrm{T}^2 + 660\tau_\mathrm{A}\tau_\mathrm{T}^3 + 360\tau_\mathrm{T}^4\right)}{(60(\tau_\mathrm{A} + \tau_\mathrm{T})(\tau_\mathrm{A} + 2\tau_\mathrm{T})(\tau_\mathrm{A} + 3\tau_\mathrm{T}))} +$$

$$\left. + 6\mathrm{e}^{-\left(\frac{3}{\tau_\mathrm{A}} + \frac{1}{\tau_\mathrm{T}}\right)(t-\mathrm{t}_0)} \frac{\tau_\mathrm{T}^3(\tau_\mathrm{A} + (3-\alpha\tau_\mathrm{A})\tau_\mathrm{T})}{\tau_\mathrm{A} + 3\tau_\mathrm{T}} - 6\mathrm{e}^{-\frac{2(t-\mathrm{t}_0)}{\tau_\mathrm{A}}} \alpha\tau_\mathrm{A}^2(\tau_\mathrm{A} - 3\tau_\mathrm{T})(\tau_\mathrm{A} - 2\tau_\mathrm{T}) \cosh\left(\frac{t-\mathrm{t}_0}{\tau_\mathrm{A}}\right) \right);$$

Eq. S1b

where F_{Spike} is the theoretical time course of the spike, t is the time elapsed from the start of the voltage stimulus, y_0 is the normalized fluorescence before the spike, t_0 is the latency of the

onset of calcium spike, F_M is the maximal normalized fluorescence increase in the absence of release termination, α is a proportionality factor [1], and τ_A and τ_T are the time constants of spike activation and termination, respectively.

Algorithms and methods used

The “Nelder-Mead simplex” algorithm was used in the MATLAB function “fminsearchbnd” [2]. The remaining algorithms were implemented using the Curve fitting toolbox and the "Nonlinear Least Squares" method of the fit object with the Algorithm property set to “Trust-Region”, “Levenberg-Marquardt” or “Gauss-Newton” and the Robust property for minimization of the influence of outliers set to “OFF” (disabled), “LAR” (least absolute residuals), or “Bisquare” (the weight in the minimized sum of squares given to each data point depends on how far the point is from the fitted line).

Both, constrained and unconstrained fitting was tested. For unconstrained fitting, the upper and lower bounds of all parameters were set to positive and negative infinity. For constrained fitting, the lower and upper bounds were [1,0, 0, 1, 1, 0] and [1,∞, ∞, ∞, ∞, 1], respectively, for the parameter set [y_0,t_0, F_M, τ_A, τ_T, α]. The lower bounds of τ_A, τ_T were set to 1 to conform with the kinetics of the fluorescent indicator OG-5N and with the 0.5 ms sampling period; the lower bound of latency was set to zero, since spikes did not occur before the start of the stimulus. The initial vector of parameters was [1,4.13, 1.577, 3.13, 5.48, 1] [3] under all conditions.

REFERENCES

1. Song LS, Sham JS, Stern MD, Lakatta EG, Cheng H (1998) Direct measurement of SR release flux by tracking 'Ca2+ spikes' in rat cardiac myocytes. JPhysiol(Lond) 512: 677-691.

2. D’Errico J (2012) Bound constrained optimization using fminsearch. MATLAB Central File Exchange: http://www.mathworks.com/matlabcentral/fileexchange/8277.

3. Zahradnikova A, Jr., Polakova E, Zahradnik I, Zahradnikova A (2007) Kinetics of calcium spikes in rat cardiac myocytes. J Physiol 578: 677-691.
