# Supplementary material for: Quantitative Analysis of Calcium Spikes in Noisy Fluorescent Background
Source: PLoS One. 2013 May 31;8(5):e64394. doi: 10.1371/journal.pone.0064394 (PMC3669300; doi:10.1371/journal.pone.0064394)
Supplement: Software Description S1 — Operation of the SpikeAnalyzer program. Screenshot S1 The Analyzer dialog window. Screenshot S2 The Fit Results Summary window. Screenshot S3 The Fitter dialog window. Screenshot S4 The FitParameters dialog window. (PDF) [file pone.0064394.s003.pdf]

Quantitative analysis of calcium spikes in noisy fluorescent background

Radoslav Janicek[1], Matej Hotka[1,2], Alexandra Zahradníková, Jr. [1], Alexandra Zahradníková[1], and Ivan Zahradník[1].

[1]Institute of Molecular Physiology and Genetics, Slovak Academy of Sciences, Vlárska 5, 833 34 Bratislava, Slovakia, [2]Department of Biophysics, Faculty of Natural Sciences, Pavol Jozef Šafárik University, Košice, Slovakia

Operation of the SpikeAnalyzer program

SpikeAnalyzer is software developed to aid analysis of calcium spikes in images recorded by the laser scanning fluorescence confocal microscopy. The images should be recorded in x-t line scan mode and saved in the TIFF format. If recorded with the Leica AOBS SP2 confocal microscope and pClamp software, the headers of these data files can be used for configuring the analysis.

SpikeAnalyzer is a Windows standalone application that uses the MATLAB Compiler Runtime (MCR) runtime engine. MRC is deployed with the executable file of this program. The scheme of data processing is depicted in the flow chart of the program (Diagram 1). The program allows analysis of multiple calcium spikes identified by the user in the loaded image. The automatic fitting procedure needs the initial estimates of the number spikes in the fluorescence trace, and of the amplitude and time of their peaks for correct initialization. This is achieved by filtering the traces using the MATLAB function `smooth` and by finding all local maxima of the smoothed trace using the MATLAB function `findpeaks`. Automatic identification of the number of peaks (0, 1, or 2) is controlled by four parameters (`Trace criteria`) specified by the user for the whole image:

i) The `amplitude threshold` - determines the minimal amplitude of a second largest fluorescence increase relative to the amplitude of the largest signal that has to be reached to consider it as a potential second spike. In other words, fitting of a second peak is attempted only if its maximum is higher than the product of the `amplitude threshold` and the smoothed maximum of the trace. The allowed values of the `amplitude threshold` are from the interval $\left(0,1\right\rangle$.
ii) The `SNR threshold` - determines the minimal allowed signal-to-noise ratio (SNR). SNR is expressed as the ratio of peak amplitude of the smoothed trace to the RMS value of the noise of the prepulse region of the trace. Fitting is attempted only if the maximum of the smoothed trace is higher than the product SNR*RMS. The allowed values of the SNR threshold are from the interval $\left\langle 1,\infty\right)$.
iii) The `smoothing factor` - controls the precision with which the smoothed trace follows the raw data. The allowed values are from the interval $\left(0,1\right\rangle$.

iv) The `maximum number of peaks` per trace to be fitted in the whole image (1 or 2).

The two fitting algorithms that provided the best estimates of spike parameters, namely, `Trust Region` and `Simplex`, are implemented as user-selectable. The fitting is based on the theoretical function describing the calcium spike. When a single peak in the fluorescence profile passes the criteria, the traces are fitted by Eq. S1 (Methods S1). When two peaks pass the criteria, a simple sum of two theoretical functions [1] is used for fitting:

$$\frac{\Delta F(t)}{F_0} = F_{Spike}\left(t, F_{M1}, \alpha_1, t_{01}, \tau_{A1}, \tau_{T1}\right) + F_{Spike}\left(t, F_{M2}, \alpha_2, t_{02}, \tau_{A2}, \tau_{T2}\right), \qquad \text{Eq. S2}$$

where the parameters corresponding to the first and the second spike are denoted by the indexes 1 and 2, respectively. A fit by Eq. S2 is accepted only if it provides a significantly better or equal goodness of fit than the fit with Eq. S1, and the fit by Eq. S1 is accepted only if it is significantly better than the fit by a constant. Since more than two calcium spikes have not been observed [1], more than two instances of Eq. S1 were not implemented into the automatic fitting routine.

The user interface of SpikeAnalyzer consists of four main windows called `Analyzer` (Screenshot 1), `Fit Results Summary` (Screenshot 2), `Fitter` (Screenshot 3), and `Fit Control` (Screenshot 4). The program starts in the `Analyzer` window. In its `File` menu there are the following options:

1) `Open` serves for loading the image (TIFF format) for analysis.
2) `Import ABF` serves for automatic configuration of the analysis by loading the relevant electrophysiological data in the pClamp format (ABF 2.0) using the functions `abf2load` [2].
3) `Configure` serves for manual setting of the analysis parameters when the image heading does not contain the information on confocal scanning frequency in the Leica TIFF format or when the information on the timing and sampling of the voltage stimulus are not available in the pClamp ABF format.
4) `Load previous project (XLS format)` loads an XLS file previously created by the SpikeAnalyzer. It serves for inspection, completion or repair of an unfinished or unsatisfactory analysis. All information necessary to continue the analysis is read from the XLS file. The list of spikes, including their positions can be updated or modified but fitting of all spikes is performed from the beginning to ensure equal conditions.

The primary analysis in the `Analyzer` window (Screenshot 1) consists of the following interactive steps:

1) Specification of the blank region (Blank ROI) for calculation of the mean background non-cell fluorescence value.

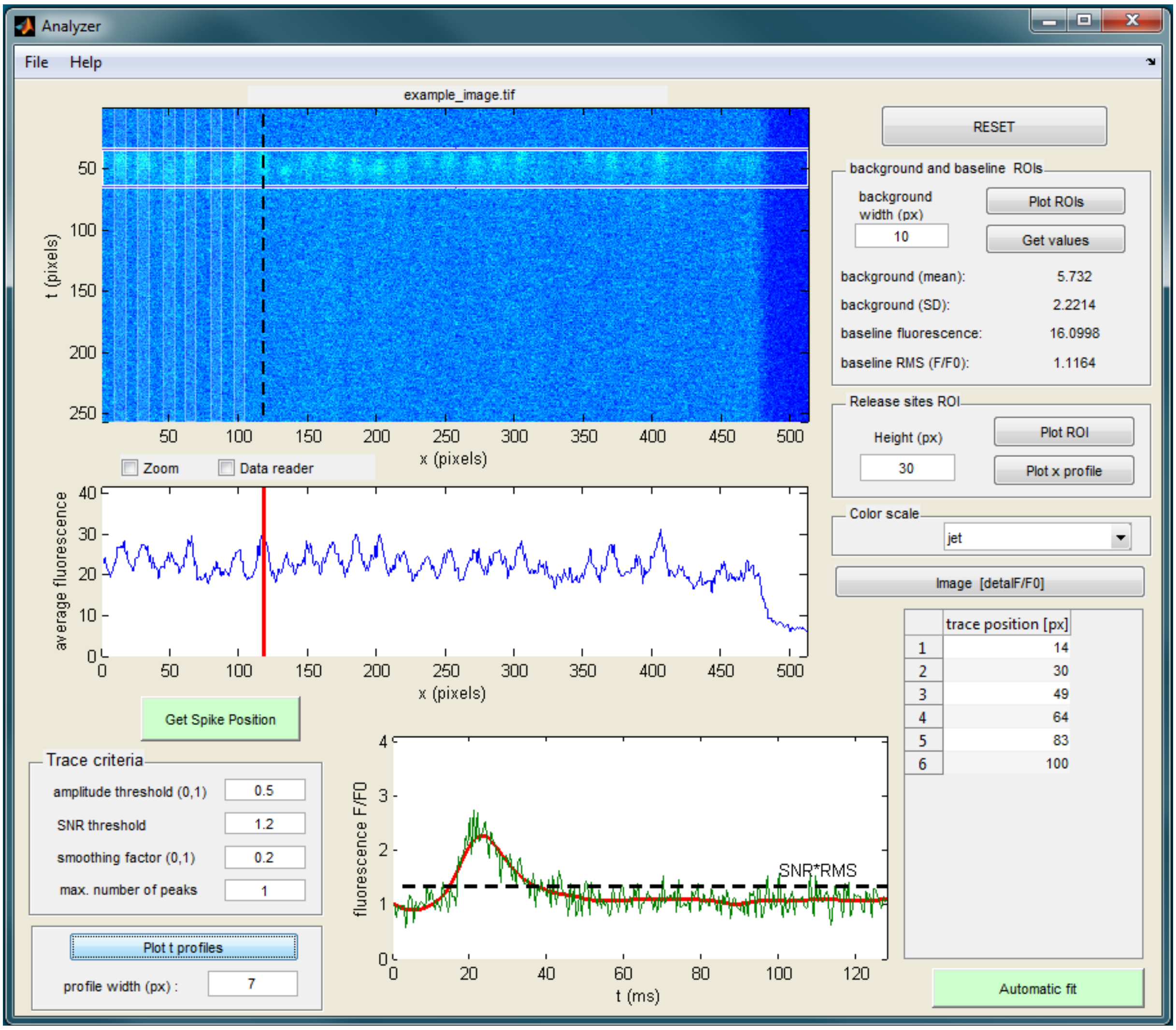

Screenshot 1. The Analyzer dialog window. The selected stripes (7 pixels wide by default) are delimited by the vertical lines in the uppermost panel. The dashed line in the uppermost panel and the red line in the middle panel show the position of the cursor, around which the average (bottom panel) is calculated.

2) Specification of the baseline region (Baseline ROI) using the stimulus information and further manual modification. The RMS noise value is automatically calculated and displayed in the `background and baseline ROIs` panel. This step is optional if the analysis of a previous project is resumed.
3) Adjustment of the region that contains calcium spikes in the `Release sites ROI` input panel. The resulting spatial profile of the calcium signal is displayed as the blue trace in the central panel using the `Plot x profile` button.
4) Identification of the positions of spikes in the profile using the red cursor and their confirmation by the `Get Spike Position` button. This step is optional if the analysis of a previous project is resumed.
5) Initialization of parameters for automatic fitting. This step is aided by the bottom panel of the `Analyzer` window, where the selected normalized fluorescence trace (green line) together with the smoothed trace (red) and the current value of SNR threshold (grey dashed line) are displayed.

6) Setting the `Trace criteria`.

The requested steps of the set-up process are indicated by the shaded buttons in the `Analyzer` window.

After identifying spikes and setting up proper fit criteria, the `Automatic fit` button, which performs fitting of all selected traces and opens the `Fit Results Summary` and `Fitter` windows shown in Screenshots 2 and 3, is enabled.

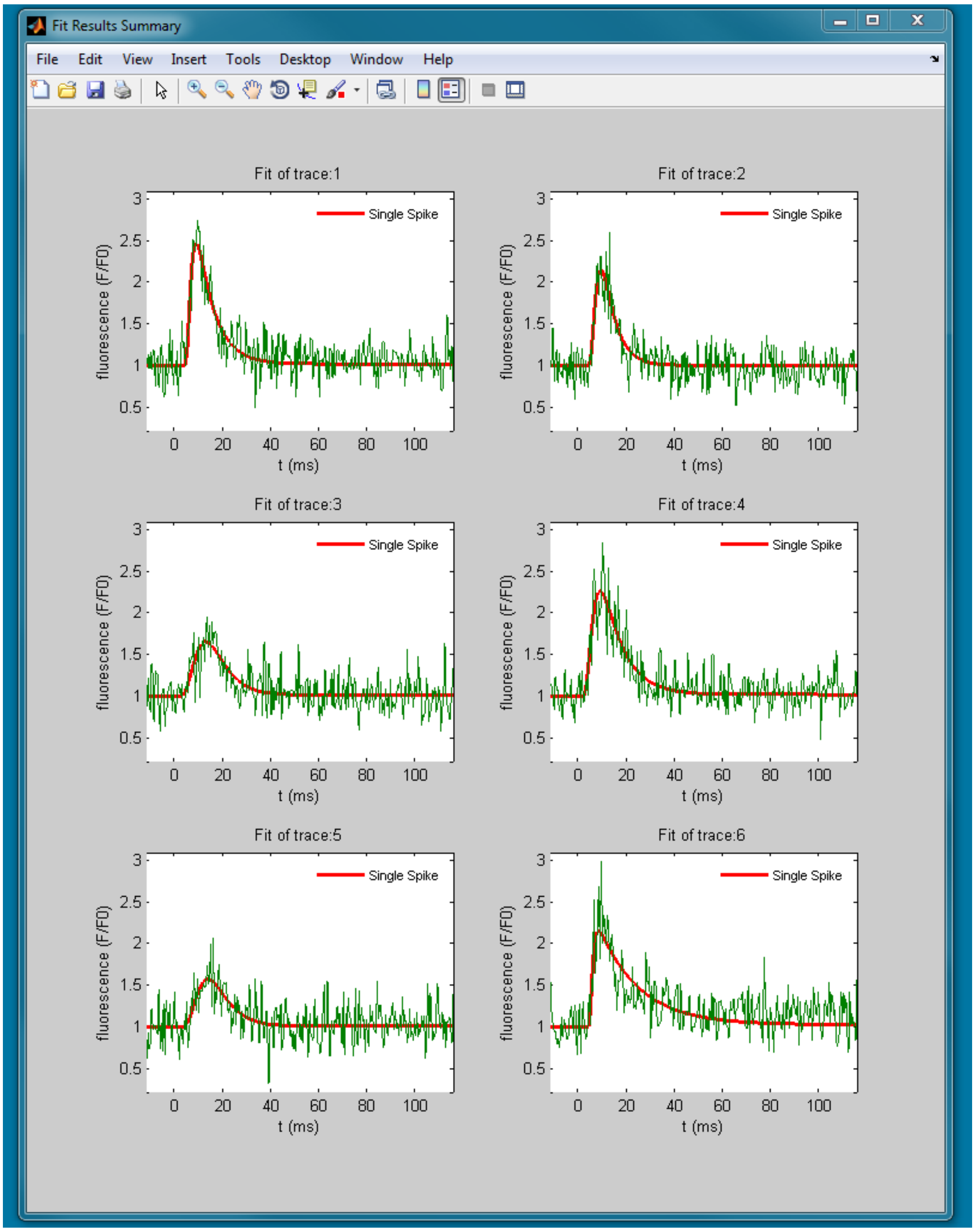

Screenshot 2. The Fit Results Summary window. Yellow panels are the repaired fits; grey panel signals that the peak amplitude of smoothed trace was smaller than product of SNR and RMS values.

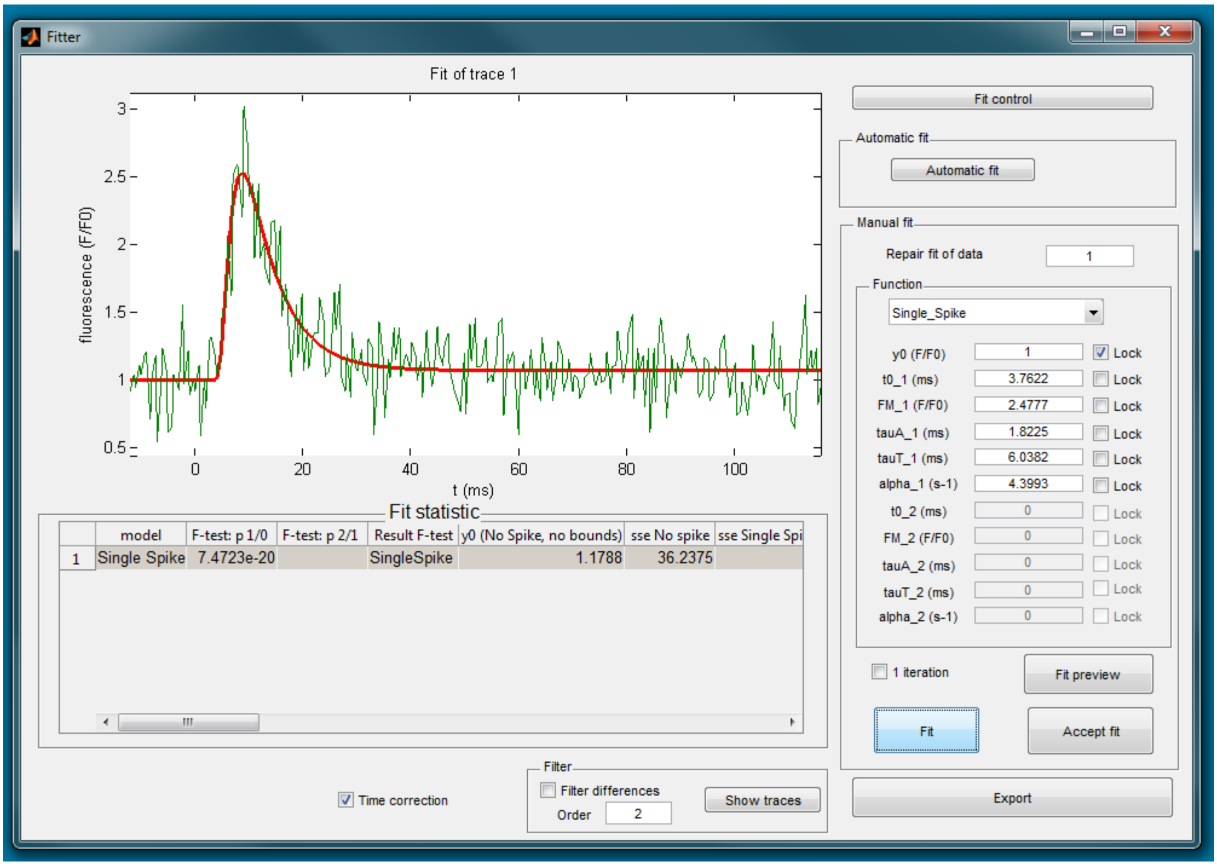

Screenshot 3. The `Fitter` dialog window.

After the automatic fit is performed, the results are displayed in the `Fit Results Summary` window (Screenshot 2). Unacceptable results can be improved by re-fitting the respective traces manually. In the `Fitter` window, the trace to be re-fitted is displayed in green, the fit in red, and the fitted parameters are shown in the panel `Function`. The user can change the fitting function (Eq. S1 for a single spike, Eq. S2 for two spikes, or a constant if a spike is not well identified in the fluorescence profile), lock parameters at the desired values, or use a filter to partially reduce noise by reducing the values of outliers. A dialog window opened by the `Fitter control` button (Screenshot 3) is used for setting the fitting algorithm and the constraints of the fitted parameters. One of four preset standard configurations of parameter constraining can be selected, or the lower and upper bounds can be specified. The user can select either independent constraints for τ_A and τ_T, or constrain τ_A and the ratio $R = \tau_A/\tau_T$. The second choice helps to find a better solution for traces that are well fitted with τ_T faster than τ_A what may not be acceptable.

The results of fitting by different functions are displayed in the Fitter window in the table `Fit Statistics` (Screenshot 2), which displays the model used; the result of the F-test; SSE (sum of squares due to error), rsquare (coefficient of determination), rmse (standard error), number of iterations; and the parameters provided by the fit. Acceptance of the fitted parameters is confirmed by clicking the `Accept fit` button (Screenshot 2).

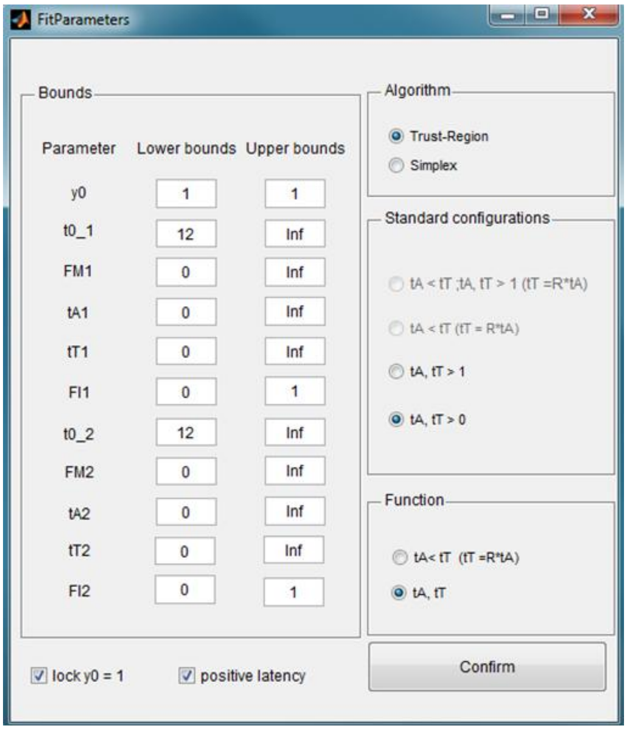

Screenshot 4. The `FitParameters` **dialog window.**

Clicking the `Automatic fit` button in the `Fitter` window starts the fitting process with the updated parameters. The results are exported using the `Export` button (Screenshot 2). All parameters of analysis, including the spike traces and the corresponding fitted traces are saved as an XLS project with the same name as that of the confocal image. Additionally, the results displayed in the Automatic fit window (Screenshot 4) are saved as a figure in the PDF format.

REFERENCES

1. Janicek R, Zahradnikova A, Jr., Polakova E, Pavelkova J, Zahradnik I, et al. (2012) Calcium spike variability in cardiac myocytes results from activation of small cohorts of ryanodine receptor 2 channels. J Physiol 590: 5091-5106.

2. Collman F. (2012) abf2load. MATLAB Central, File Exchange, http://www.mathworks.com/matlabcentral/fileexchange/22114-abf2load. Retrieved August 21, 2012.
